# An efficient hexadecimal network flow watermark method for tracking attack traffic

**DOI:** 10.1038/s41598-023-48552-0

**Published:** 2023-11-30

**Authors:** Jun Cui, Keya Han, Lin Sha, Wei Liu, Xiaofeng Zhang, Guangxu Li

**Affiliations:** 1 School of Life Sciences, Tiangong University, Tianjin, 300387 China; 2 School of Control Science and Engineering, Tiangong University, Tianjin, 300387 China; 3 School of Electronics and Information Engineering, Tiangong University, Tianjin, 300387 China

**Keywords:** Computer science, Information technology, Software

## Abstract

Network flow watermark technology is a traffic marking technique that embeds watermark information into the characteristics of network flows to mark and trace attack flows generated by network attackers. However, with the development of network attack techniques, the time and number of packets required for network attacks have decreased. Existing network flow watermark technologies fail to balance watermark robustness and efficiency, resulting in poor practicality. To address this issue, this paper proposes an efficient hexadecimal network flow watermark method. The method introduces an efficient interval watermark algorithm and utilizes an interval synchronization algorithm to self-learn watermark parameters, thereby improving the encoding efficiency of the watermark. The design of watermark start and end markers ensures the practicality of network watermarks, enabling traceability and source attribution of attack flows in real network environments. The proposed method is experimentally tested using real network traffic, and the results demonstrate that even in the presence of a network jitter, the watermark detection success rate of this scheme remains above 95%. Compared to other network flow watermark schemes, the hexadecimal network flow watermark proposed in this paper achieves a 50% improvement in encoding and decoding efficiency while ensuring robustness. It also exhibits excellent resistance to network jitter, packet loss, and false packet insertion.

## Introduction

With the rapid development of the internet, we have entered the era of connected everything. While the internet has brought many conveniences, it has also created many problems, with Internet security being one of the most serious. Incidents of network fraud, privacy leaks, and the emergence of various network attack methods have increased sharply, leading people to realize the importance of network security. Intrusion tracking technology^1^ has emerged as a way to discover the real location of network intruders and take appropriate countermeasures against intrusion behavior. However, attackers often use stepping stones^2–4^ and anonymous protocols^5–7^ to hide their real IP addresses during attacks, making intrusion tracing extremely difficult. Stepping stones are intermediate hosts that forward attacker traffic to another remote destination address, which can mask the true origin of the attack and make intrusion tracing more challenging.

In recent years, various traffic analysis techniques have been developed to track attack traffic through stepping stones. The traditional method is passive traffic analysis^8–10^, which uses statistical methods to find correlations between outbound and inbound traffic. However, these methods require long observation times and cannot work in real-time because their computational costs are high when processing a large amount of collected traffic, and they are also highly susceptible to network jitter. Active traffic analysis technology^11, 12^ is another method, with network flow watermark (NFW) technology being the mainstream. NFW marks and traces network traffic by adjusting certain features of network flows. The embedding and detection sides share some watermark parameters. The embedding side embeds the watermark signal into the incoming traffic, and the detection side decodes these watermark signals from the outgoing traffic to discover the correlation of traffic. Compared with passive traffic analysis, NFW requires fewer data packets and is more suitable for traffic tracing^13^. Due to its high precision, low resource consumption, concealment, and adaptability, active network flow watermark has become one of the mainstream methods for traffic tracing.

According to the type of carrier, NFW can be divided into network flow watermark based on flow rate^12–15^, inter-packet delay^16–22^, and time slot interval^23–31^. Among them, time-based network flow watermark has better concealment and has become the research focus of network flow watermark in recent years. Time-based NFW can be further divided into three types: inter-packet delay-based, interval packet count-based, and interval centroid-based. Many advanced interval centroid watermark schemes sacrifice other performance aspects, such as watermark efficiency, in exchange for robustness. Although this improves the robustness of the watermark, there is a problem of low watermark efficiency. The watermark requires a large time overhead and often needs to cache thousands of packets to embed the watermark, while attackers can complete network attacks on the target in a very short time. Therefore, the length of the target network flow is often short, resulting in poor practicality of the watermark. The key challenge in designing an efficient NFW scheme is to ensure the robustness of the watermark while maximizing the watermark encoding efficiency as much as possible.

In this study, we propose a concept of “watermark capacity” to characterize the encoding efficiency of network flow watermark. To improve the watermark capacity, we propose an efficient hexadecimal NFW scheme based on an adaptive interval length. Unlike other existing watermark schemes, the proposed NFW scheme adapts to the interval length $T$ by collecting data stream IPD features and then dividing an interval into six sub-intervals. Watermark data is embedded by delaying packets to change the number of packets in the sub-intervals. The scheme modulates 4-bit watermark data in an interval to improve watermark encoding efficiency and increase the watermark capacity in the network flow.

The main contributions of this research are as follows:

1. We propose the concept of “watermark capacity”, which refers to the number of watermark bits that can be embedded in a fixed-rate network flow within a unit of time. This concept is used to characterize the encoding efficiency of network flow watermark schemes.
2. We propose an efficient hexadecimal network flow watermark scheme with adaptive interval length. This scheme can self-learn watermark parameters based on network traffic features and improves watermark encoding efficiency. It can embed more watermark bits in a finite-length network flow while ensuring watermark robustness, thereby increasing the watermark capacity of the network flow.

The remaining parts of this paper are organized as follows: In “Related work” section, we introduce several state-of-the-art NFW methods and related works on NFW. In “Methodology” section, we elaborate on the theoretical scheme of the proposed NFW scheme, including the concept of watermark capacity, the adaptive interval length algorithm, and the efficient hexadecimal encoding and decoding methods for network flow watermark. The experimental results and analysis are presented in “Experimental results and analysis” section. Finally, in “Conclusion and future work” section, we summarize the conclusions of this paper and suggest future work.

## Related work

In recent years, various scholars have proposed different methods for network flow watermark (NFW) to detect stepping stones^18^, tracking anonymous communication systems^26^, and cloud track^32, 33^. This article examines the current mainstream NFW technologies and categorizes them into three types based on the carrier of the watermark signal: rate-based NFW, inter-packet delay-based NFW, and slot-based NFW.

### NFW based on flow rate

Flow rate refers to the transmission rate of network traffic within a specific period. NFW technology based on flow rate modulates the data flow rate at specific times to embed watermark information, while collecting the flow rate extracts watermark information to track data flow. A typical method proposed by Yu et al. in 2007^14^, called DSSS-W, spreads the original signal through Pseudo-Noise(PN) codes at the sender and slightly adjusts the flow rate during the spreading period to embed the watermark using a certain adjustment amplitude. This method can accommodate numerous watermarks, track multiple flows in parallel, and achieve a high tracking rate in a stable flow rate environment. However, it is not suitable for networks with large fluctuations in flow rate. Fu et al.^12^ transformed the time-domain watermark into a feature-invariant frequency using frequency-domain analysis technology and embedded it into wireless network data flows through electromagnetic interference, effectively reducing the anonymity of flow-based wireless Mix networks. In 2011, Huang et al.^15^ improved Fu's scheme, which can effectively resist Mean Square-related attacks. However, the scheme's applicability is poor due to difficulties in generating long PN codes that satisfy the conditions.

### NFW based on inter-packet delay

Inter-packet delay (IPD) is the time difference between the arrival or departure of a pair of packets in the same network flow. NFW based on IPD changes the IPD between packets by delaying certain packets in the network flow to embed watermark signals. In 2003, Wang and Reeves^16^ proposed an IPD-based watermark scheme, in which several packets in a short network flow are randomly selected, and the watermark signal is embedded by fine-tuning the IPD of the packets. This approach has the advantages of simple implementation and high accuracy but cannot be used to track real-time flows. To address these issues, Wang et al.^17^ proposed another IPD-based network flow watermark method in 2005. This technique does not quantify the IPD of network flows but changes the average value of a set of normalized IPD differences by adjusting the incremental parameter to embed the watermark signal.

In 2007, Park and Reeves^18^ proposed an adaptive network flow watermark technique that adaptively selects watermark parameters based on the actual situation of the watermark network flow to complete the watermark embedding. In 2009, Pan et al.^19^ proposed an IPD analysis attack idea based on adjacent "footprints" and gave an algorithm to infer watermark parameters, which can significantly alleviate the decrease in watermark detection rate caused by time perturbation. To reduce the impact of adding virtual packets to network flows, Peng et al.^20^ used packet matching technology in their watermark scheme. This approach matches appropriate packets in network flows and associates data flows, which can reduce the impact of fake packets on the watermark. To analyze flow correlation using non-blind watermark and achieve good robustness against attacks, Houmansadr et al.^21^ proposed a non-blind watermark scheme called RAINBOW. They added an IPD database to the watermark framework for storing IPD. In 2018, Iacovazzi et al.^22^ proposed an invisible network flow watermark technique called DropWat for data leakage attacks.

### NFW based on time slot intervals

The NFW based on time slot interval achieves watermark embedding and detection by modulating the number of packets or the centroid value of packets within a data flow interval. However, flow watermark based on inter-packet delay (IPD) cannot work effectively when packet loss or packet reordering occurs. To solve this problem, Pyun et al.^23^ proposed the first interval-based watermark (IBW) scheme in 2007. In the IBW scheme, the network flow is divided into multiple equal-length periods from a certain offset, with each period being a time slot interval. Watermark embedding is achieved by manipulating the number of packets in the interval through delayed packets, which can solve packet disturbance and packet grouping problems. In 2012, Pyun et al.^24^ proposed an improved IBW scheme with self-synchronization property, which can effectively solve problems such as fake packets, flow segmentation, and flow merging. In 2021, Mo et al.^25^ proposed an efficient quaternary network flow watermark technique, which can embed 2-bit watermark data within an interval and improve the coding efficiency of the watermark to some extent by interval division.

Wang et al.^26^ proposed the first centroid-based watermark scheme (ICBW) in 2007. The centroid of an interval is defined as the average offset of each packet arrival time relative to the start time of the interval. Watermark information is embedded by delaying packets to change the centroid value. In 2010, Wang et al.^27^ proposed a double-centroid-based watermark scheme (DICBW), which selects two adjacent intervals to embed the watermark, resulting in a higher detection rate. To better defend against multi-flow attacks (MFA), Luo et al.^28^ combined ICBW with direct sequence spread spectrum (DSSS) coding technology and proposed a centroid-based spread-spectrum watermark scheme (ICBSSW) in 2012. The scheme can effectively resist MFA and is suitable for parallel tracking of multiple attack flows. However, watermark detection in ICBSSW requires repeated parameter attempts, which leads to high time and space costs.

To enhance the stealthiness of flow watermark, Liu et al.^29^ proposed a high-stealth NFW scheme based on centroid using an internal swapping algorithm in 2018. The interval centroid is changed by exchanging a limited number of IPDs within the time slot interval, without changing the time characteristics of the network flow, which improves the scheme's stealthiness. Hou et al.^30^ proposed a watermark technology based on histogram-specified centroid intervals, which improves the resistance of existing technology to multi-flow attacks in edge computing and reduces the time and space costs of the detector. LIU Kexian et al.^31^ proposed a multiple redundant flow fingerprint model based on time slots in 2023. Operating data packets within specified time slots to embed fingerprints can effectively detect man-in-the-middle attacks and prevent network identity spoofing.

Through analysis of existing NFW technology, it is found that advanced NFW schemes have good robustness and concealment. However, the disadvantage is that the watermark encoding efficiency is low, resulting in a small watermark capacity for network flows, which cannot track some shorter network traffic and has poor practicality. Therefore, in this study, we designed an efficient hexadecimal NFW scheme with adaptive interval length. This scheme can self-learn watermark parameters based on network flow characteristics and improve the watermark encoding efficiency, thereby enhancing the watermark capacity of network flows and improving the practicality of watermark. It can ensure good robustness for different types of network traffic.

## Methodology

In this section, we describe in detail the watermarking capacity, the overall framework of watermark and the hexadecimal network flow watermark scheme.

### Watermark capacity

In various network scenarios, significant differences exist in network flow rate, length, number of packets, and other aspects. In some practical network scenarios, the network watermark needs to be embedded in relatively short network flows, which requires the watermark to have high coding efficiency and embed sufficient watermark information in the shortest possible network flow while ensuring the watermark's accuracy. This paper proposes using the watermark capacity of the network flow to characterize the watermark's coding efficiency.

Assuming that the packet throughput rate of the network flow is stable, the formula for calculating the watermark capacity as Eq. (1).

1 $$S = V_{pps} \times \frac{B}{P}$$

In the formula, $V_{pps}$ represents the packet throughput rate of the network flow, which characterizes the packet flow rate of the network flow and is measured in pps, indicating how many packets are sent per second. $B$ represents the number of embedded watermark bits, $P$ represents the number of packets required to embed $B$ bits of watermark information, and the watermark capacity $S$ is measured in bits per second (bit/s).

The watermark capacity represents the number of watermark bits that can be transmitted in a unit of time in the network flow, i.e., the watermark-carrying capacity of the network flow, which directly reflects the coding efficiency of the watermark scheme.

### Watermark overall framework

**Figure 1:**
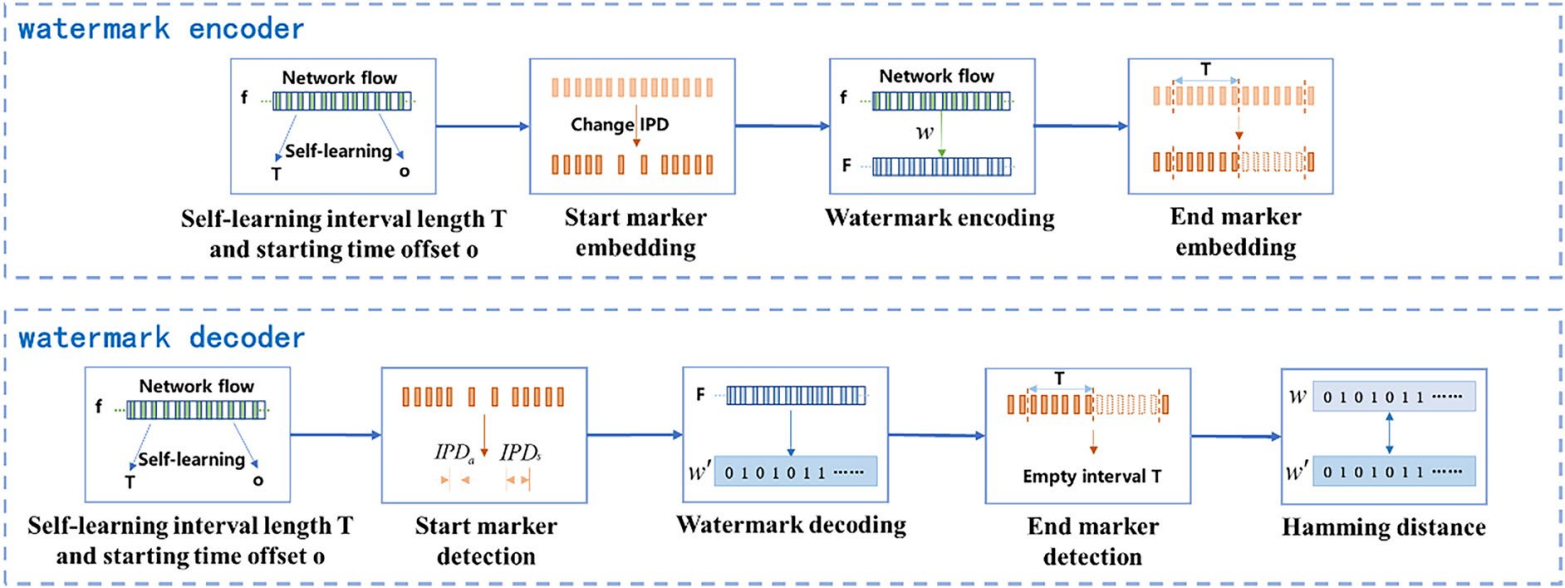
The overall framework of the watermark scheme.

For different application scenarios of network flow watermark, we have found that the characteristics of network flows differ greatly. Existing network flow watermark technologies cannot dynamically adjust watermark parameters, resulting in low coding efficiency of watermark, small watermark capacity of network flows, and poor practicality of network flow watermark. In order to further enhance the watermark capacity of network flows and improve the practicality of flow watermark, this paper proposes an efficient hexadecimal network flow watermark method based on the efficient quaternary network flow watermark scheme^25^. We combine it with the watermark interval synchronization algorithm to further improve the coding efficiency and practicality of the scheme. The overall framework of the watermark scheme is shown in Fig. 1.

To achieve slot interval synchronization, the watermark embedding and detection parties first learn the network flow characteristics and adaptively synchronize the interval length $T$ and the starting offset time $o$ before starting the watermark transmission to achieve interval synchronization between them. After the slot synchronization is completed, the embedding party delays sending data packets to embed the watermark start marker, and then encodes the watermark bit sequence $w$ into the network flow. Once the encoding is completed, the embedding party generates an empty interval $T$ without sending any data packets to embed the watermark end marker.

The watermark detection party receives the network flow, self-learns the interval length $T$ and the starting offset time $o$, and then detects the watermark start marker. After detecting the start marker, the detection party prepares to receive the watermark bit information, determines the interval position based on the self-learned interval length $T$ and starting offset time $o$, and then decodes the watermark bit sequence $\dot{w}$ from the network flow until the watermark end marker is detected, which is an empty interval $T$ with no data packets arriving. After detecting the watermark end marker, the Hamming distance between the watermark bit sequences $w$ and $\dot{w}$ is calculated to verify whether the embedded watermark information is detected.

This scheme mainly includes adaptive interval synchronization, watermark start and end markers, hexadecimal watermark scheme, and watermark decoding. These components will be described in detail below.

### Hexadecimal NETWORK FLOW WATERMARK SCHEme

In this section, we describe in detail the interval synchronization algorithm, watermark start and end marker, watermark encoding and watermark decoding.

#### Interval synchronization algorithm

Before embedding the watermark, it is necessary to determine the watermark parameters (including the n-bit watermark bit sequence $w = \left\{ {w_{1} ,w_{2} , \ldots ,w_{n} } \right\}$, interval length $T$, and starting offset time). These parameters should be consistent between the watermark embedding and detection sides. To efficiently embed and detect watermarks in different network flows, this scheme only shares the watermark bit sequence $w$ between the watermark embedding and detection sides, and other parameters are dynamically self-learned through adaptive network flow features. Before embedding the watermark, the watermark embedding and detection sides simultaneously collect network flow features (network flow packet throughput and packet fluctuation), and synchronize the interval length $T$ and starting offset time $o$ through the adaptive interval algorithm, realizing interval synchronization between the watermark embedding and detection sides.

The method of calculating the interval length $T$ and starting offset time $o$ by the interval synchronization algorithm as follows:

2 $$T = 12\left( {\frac{1 + \sigma }{{V_{pps} }}} \right)$$

3 $$o = 2T$$

Here, $V_{pps}$ represents the throughput of the data packets in the network flow, and $\sigma$ is the standard deviation of the inter-packet delay (IPD), which reflects the degree of fluctuation of the data packets in the network flow. The calculation method is as Eq. (4).

4 $$\sigma = \sqrt {\frac{{\mathop \sum \nolimits_{i = 1}^{k} \left( {d_{i} - d_{a} } \right)^{2} }}{k}}$$

The IPD is the time difference between two adjacent packets of the target network flow when they arrive at the host. $d_{i}$ represents the IPD of the i-th packet in the network flow starting from the interval synchronization, and $d_{a}$ represents the average IPD of $k$ packets. $d_{a} = \mathop \sum \limits_{i = 1}^{k} d_{i} /k$.

Using the Adaptive Interval Length Algorithm, it is possible to achieve adaptive watermark parameter tuning for various network environments. This approach maximizes the transmission efficiency of network watermarks while ensuring data reliability, significantly enhancing the efficiency of network flow watermarking.

#### Watermark start and end markers

**Figure 2:**
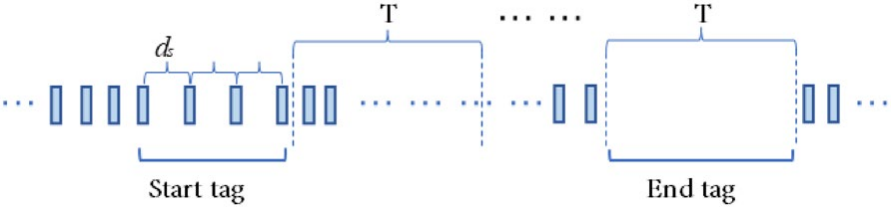
The theoretical model of watermark start and end flags.

To enhance the practicality of the watermark scheme, this study introduces a watermark start marker at the beginning of the watermark and a watermark end marker at the end of the watermark to accurately locate the watermark position and improve the efficiency and practicality of the network flow watermark. The theoretical model of the watermark start and end markers is shown in Fig. 2. After the watermark embedding party learns the average IPD $d_{a}$, the interval length $T$, and the starting offset time $o$, it uses three consecutive IPD $d_{s}$ as the watermark start marker, where $d_{s} = 3d_{a}$ represents the first IPD in the sequence. After encoding the watermark, the watermark embedding party delays sending data packets to generate an empty interval $T$ without sending any data packets and embeds the watermark end marker.

After self-learning the average packet inter-arrival time $d_{a}$, interval length $T$, and starting offset time $o$, the watermark detection party continues to learn the network flow characteristics and records the IPD value $d_{i}$. It monitors the arrival of the watermark start marker and if three consecutive IPD values $d_{i}$ are all consistent with $2.5d_{a} \le d_{i} \le 4.5d_{a}$, it indicates the detection of the watermark start marker, and the watermark detection party starts to decode the watermark bit information. Based on the distribution of data packets within the interval $T$, the watermark detection party decodes the watermark bit. If no packets arrive in interval $T$, it indicates the detection of the watermark end marker, and the watermark detection party ends the watermark bit decoding. The watermark detection process is then completed.

#### Watermark encoding

This paper proposes an efficient hexadecimal network flow watermark method that improves upon the existing efficient quaternary network flow watermark method. The proposed scheme divides the network flow into multiple time slots $I_{i}$ of length $T$ starting from an initial offset $o$, which is automatically obtained through the interval synchronization algorithm discussed earlier. Each time slot $I_{i}$ is further divided into six equally spaced consecutive sub-intervals, denoted as $I_{i,1}$, $I_{i,2}$, $I_{i,3}$, $I_{i,4}$, $I_{i,5}$ and $I_{i,6}$ , with the number of packets in each sub-interval denoted as $P_{i,1}$, $P_{i,2}$, $P_{i,3}$, $P_{i,4}$, $P_{i,5}$ and $P_{i,6}$, respectively. For a properly sized network flow, the interval synchronization algorithm yields appropriate values for $T$ and $o$, and the packets in each time slot $I_{i}$ are uniformly distributed, such that the number of packets in each of the six sub-intervals is almost equal. Assuming that the interval $I_{i}$ is divided into six consecutive sub-intervals, $\overline{{P_{i} }}$ is the average number of data packets in each subinterval within interval $I_{i}$,the difference between the number of packets in the first sub-interval and half of the average number of packets in the six sub-intervals as Eq. (5).

5 $$D_{i,1} = P_{i,1} - \overline{{P_{i} }} /2$$

where $$\overline{{P_{i} }} = \frac{1}{6}\left( {P_{i,1} + P_{i,2} + P_{i,3} + P_{i,4} + P_{i,5} + P_{i,6} } \right)$$.

Similarly,

6 $$D_{i,2} = P_{i,2} - \overline{{P_{i} }} /2,\;\;\;D_{i,4} = P_{i,4} - \overline{{P_{i} }} /2\;\;\;{\text{and}}\;\;\;D_{i,5} = P_{i,5} - \overline{{P_{i} }} /2$$

The data packets in $I_{i}$ are approximately uniformly distributed within the interval, so it can be inferred that $E\left( {D_{i,1} } \right)$, $E\left( {D_{i,2} } \right)$, $E\left( {D_{i,4} } \right)$, and $E\left( {D_{i,5} } \right)$ are all greater than or equal to zero.

**Figure 3:**
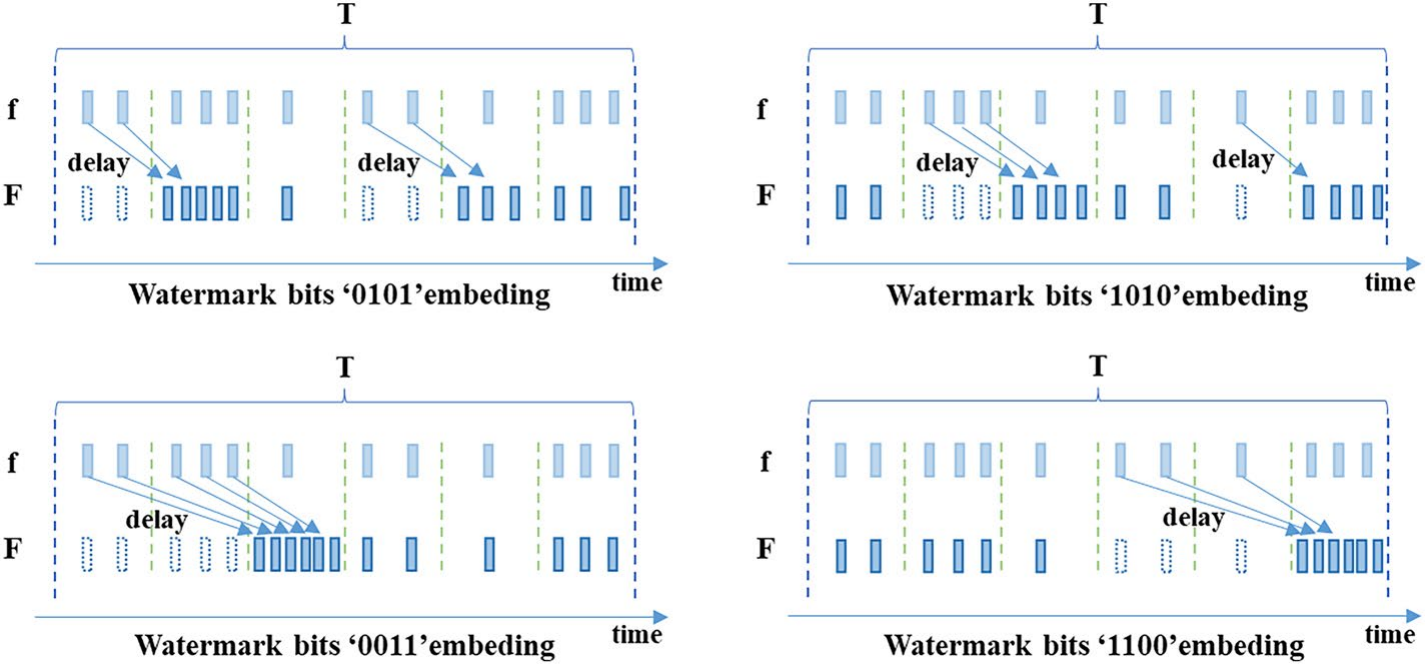
The encoding process of four-bit watermark data.

For the target network flow f, the watermark embedding scheme forms a new network flow F by delaying or directly transmitting the data packets in the interval to achieve watermark encoding of the network flow, with a delay time of $T/6$. This scheme uses six sub-intervals in one interval $T$ to encode 4-bit watermark data. By delaying the transmission of data packets and changing the number of packets in the first, second, fourth, and fifth sub-intervals of interval $I_{i}$ ($P_{i,1}$, $P_{i,2}$, $P_{i,4}$, and $P_{i,5}$), the value of $D_{i,1}$, $D_{i,2}$, $D_{i,4}$, and $D_{i,5}$ can be controlled to be greater than or less than 0 to encode the watermark data. The encoding process of four-bit watermark data is shown in Fig. 3.

#### Watermark decoding

The detection party initially learns the interval length $T$ and the starting offset time $o$ through a self-learning process, which is then recorded. Upon detecting the watermark start marker and reaching the starting offset time $o$, the first interval begins. Each interval, with a time length of $T$, is divided into six sub-intervals, which cycle every time $T$ is passed. Each sub-interval has a length of $T/6$, and the number of data packets arriving in each sub-interval is recorded and saved. At the end of the sixth sub-interval, the number of data packets in the entire interval is calculated by summing the recorded number of data packets in each sub-interval, denoted as $P_{i}$. The average number of data packets in each sub-interval, $P_{i,1}$, is then calculated, and the difference between the number of data packets in each sub-interval and $\overline{{P_{i} }} /2$ is obtained as $D_{i,1}$, $D_{i,2}$, $D_{i,4}$, and $D_{i,5}$. The watermark bit information is decoded based on the magnitude of $D_{i,k}$, The calculation method is as follows.

7 $$\dot{w}_{4i} = \left\{ {\begin{array}{*{20}c} {1, D_{i,1} \ge 0 } \\ {0, D_{i,1} < 0} \\ \end{array} } \right.\;\;\;{\text{and}}\;\;\;\dot{w}_{4i + 1} = \left\{ {\begin{array}{*{20}c} {1, D_{i,2} \ge 0 } \\ {0, D_{i,2} < 0} \\ \end{array} } \right.$$

8 $$\dot{w}_{4i + 2} = \left\{ {\begin{array}{*{20}c} {1, D_{i,3} \ge 0 } \\ {0, D_{i,3} < 0} \\ \end{array} } \right.\;\;\;{\text{and}}\;\;\;\dot{w}_{4i + 3} = \left\{ {\begin{array}{*{20}c} {1, D_{i,4} \ge 0 } \\ {0, D_{i,4} < 0} \\ \end{array} } \right.$$

Using the watermark extraction algorithm described above, the watermark information is sequentially decoded based on the distribution of data packets in the interval $T$, with 4-bit watermark data decoded in each interval of length $T$. Watermark detection is stopped when the watermark end marker is detected, which is an empty interval where no data packets arrive. Finally, the decoded watermark bit data, $\dot{w}$, is compared to the embedded watermark bit data, $w$, for similarity by calculating the Hamming distance, $H\left( {w, \dot{w}} \right)$, between the two-bit sequences $w$ and $\dot{w}$. If $H\left( {w, \dot{w}} \right)$ is less than the set Hamming distance $h$, it indicates successful detection of watermark information. Otherwise, it indicates that the watermark information has not been detected. Setting the Hamming distance instead of exact matching to determine the presence of a watermark improves the robustness of the watermark against interference from attackers and increases the success rate of watermark detection.

## Experimental results and analysis

In this section, we simulated a realistic network environment to evaluate the proposed network flow watermark scheme and compared it with two existing watermark schemes, namely DICBW^27^ and the efficient quaternary flow watermark scheme^25^. DICBW and the efficient quaternary network flow watermark scheme are two stable and efficient interval-based network flow watermark technologies. In DICBW, 1-bit watermark data is embedded by selecting an adjacent pair of intervals and adjusting the data packets within the intervals to change the centroid difference between the two adjacent intervals. The efficient quaternary network flow watermark scheme embeds watermark data by adjusting the distribution of data packets within an interval, which involves changing the number of data packets within the interval.

### Experimental environment

**Figure 4:**
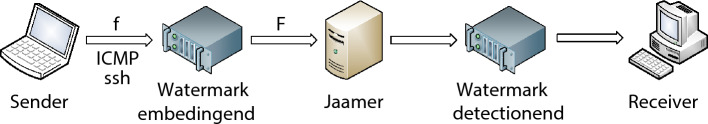
The topology of the experimental environment.

The experimental environment, built for this paper, is illustrated in Fig. 4. Five edge computing nodes, namely the sender, watermark embedding end, jammer, watermark detection end, and receiver, are connected through a local area network. The sender is responsible for generating network flows comprising of ICMP and SSH traffic, which are sent to the receiver to generate the target network flow required for watermark. Prior to the watermark process, the watermark embedding end and watermark detection end share the original watermark information $w$. In real-time, the watermark embedding end modulates the target network flow based on the characteristics of the flow, embeds the original watermark information $\dot{w}$ into the network flow, and then sends the modulated flow to the jammer. The jammer introduces jitter and random packet loss to simulate the network jitter and packet loss that may occur in a real network environment. The watermark detection end learns watermark parameters based on the characteristics of network traffic, records the arrival time of data packets in the network flow, detects watermark information $\dot{w}$, and finally calculates the Hamming distance between the detected watermark information $w$ and the original watermark information $\dot{w}$. The calculated Hamming distance determines whether the watermark is detected, thereby achieving flow correlation and completing intrusion tracking.

The network flow packets in the experiment are all real network packets, including ICMP protocol packets and SSH protocol packets, sent from the sender to the receiver. Our experimental data was chosen from the dataset provided by Cooperative Association for Internet Data Analysis (CAIDA) in February 2015.

### Watermark capacity

This paper presents a novel efficient hexadecimal network flow watermark method, and its coding efficiency is verified in comparison to the DICBW and efficient quaternary network flow watermark schemes. The experiments are conducted on ICMP and SSH network traffic to calculate the watermark capacity of the three schemes and compare their coding efficiency.

**Figure 5:**
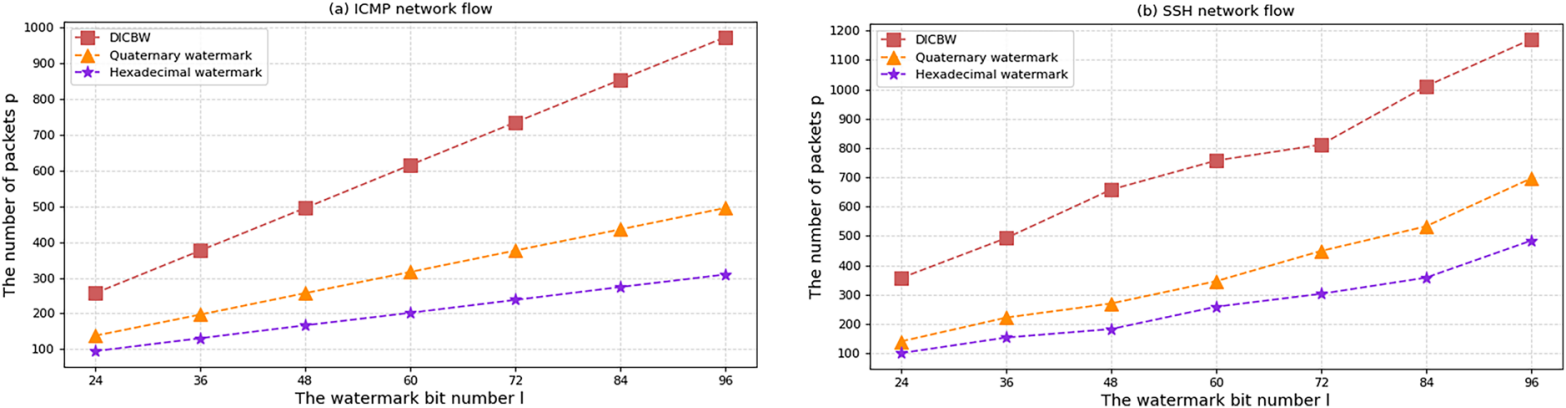
The number of packets required to embed different numbers of watermark bits.

In network flows with the same speed, embedding more watermark bits requires more data packets. The relationship between the number of watermark bits and the required data packets is experimentally verified to compare the coding efficiency of different watermark schemes. The experimental parameters are set as follows: the flow rate of the network flow data packets is $V_{pps} = 5pps$, meaning that five data packets are transmitted per second. According to the self-learning interval length algorithm proposed in this paper, the interval length of the proposed watermark scheme is determined to be from 2 to 3 s. Therefore, the interval length of the ICBW and efficient quaternary network flow watermark schemes is set to 2 s. The starting offset time is $o = 1.5\;{\text{s}}$, and the watermark bit number is increased by 12 for each experimental recording. As shown in Fig. 5a for the experimental results under ICMP network flow and Fig. 5b for the experimental results under SSH network flow, the horizontal axis represents the watermark bit number, and the vertical axis represents the number of data packets required to embed the watermark.

Under the condition of unchanged parameters, the number of data packets required to embed the watermark increases with the increase of the embedded watermark bit number l. It can be clearly seen that for the same embedded watermark bit number, the proposed efficient hexadecimal network flow watermark method requires fewer data packets than the other two schemes and has higher watermark coding efficiency. In ICMP and SSH network flows, when the Hamming distance $h$ is set to 5, the detection success rate of the three watermark schemes is above 95%.

When the packet flow rate $V_{pps} = 5pps$ and the watermark sequence is 60 bits, according to the Eq. (1) for calculating the watermark capacity, the watermark capacity $S = 1.49\;{\text{bit}}/{\text{s}}$ of the efficient hexadecimal network flow watermark scheme, the watermark capacity $S = 0.49\;{\text{bit}}/{\text{s}}$ of DICBW, and the watermark capacity $S = 0.95\;{\text{bit}}/{\text{s}}$ of the efficient quaternary network flow watermark scheme can be obtained. In the same network flow environment, the watermark capacity of the efficient hexadecimal network flow watermark scheme is the highest, about three times that of the DICBW scheme and 1.5 times that of the efficient quaternary network flow watermark scheme.

**Figure 6:**
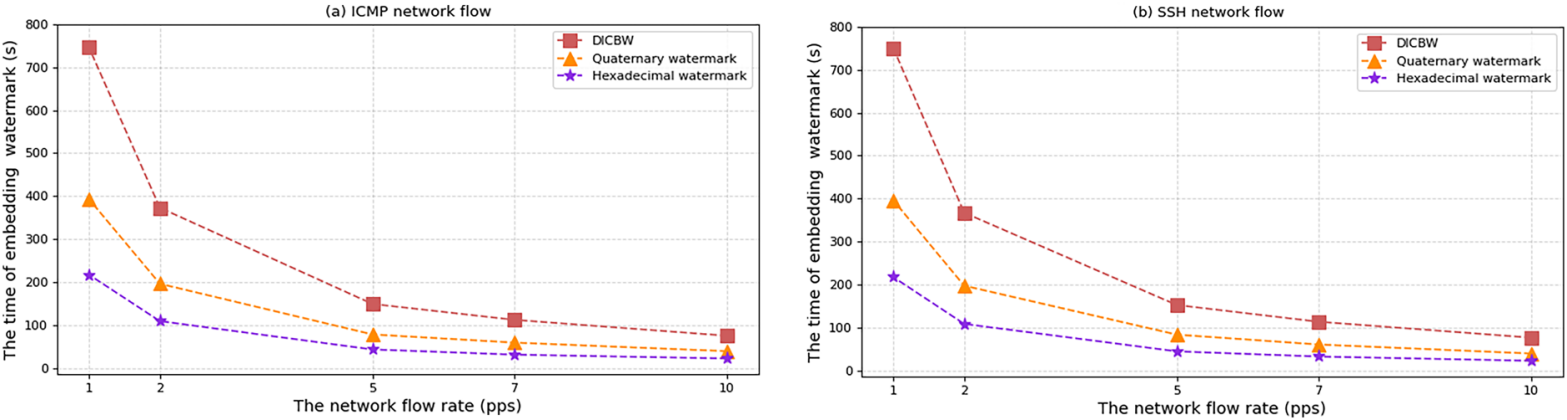
The time required to embed the watermark with different network rate.

When embedding network flow watermarks in different flow rate network flows, the time required is different. Theoretical analysis shows that when embedding the same length of the watermark bit sequence, the faster the network flow rate, the shorter the time required. The experimental parameters are set as follows: setting the starting offset time $o = 2\;{\text{s}}$, Hamming distance $h = 3$, and embedding a watermark sequence of length 60 bits in ICMP and SSH network flows, respectively. The time required for embedding a 60-bit watermark for the three network flow watermarks in different network flow rates is recorded. Figure 6a and b respectively represent the experimental results of ICMP and SSH network flows. From the figure, it can be seen that compared with the DICBW and efficient quaternary network flow watermark schemes, in the same network flow rate, the efficient hexadecimal network flow watermark scheme proposed in this paper requires the shortest time to embed the same length of the watermark bit sequence, and has the highest watermark encoding efficiency. In ICMP and SSH network flows, when the Hamming distance $h = 3$, the detection success rate of the three watermark schemes is above 95%.

The above experimental results demonstrate that, compared with the other two interval-based NFW schemes, the efficient hexadecimal network flow watermark scheme proposed in this paper has higher encoding efficiency, requires less time and fewer data packets to embed the same watermark length, and has a higher network flow watermark capacity.

### Watermark robustness

In the practical application of network flow watermark, the robustness of the watermark in actual environments is more important than the efficiency of watermark encoding. To test the robustness of watermark schemes, we conducted experimental tests on the SSH network flow. We chose an original watermark length of 60 bits and set the Hamming distance $h$. We then calculated the Hamming distance between the detected watermark sequence and the original watermark sequence. If the Hamming distance is less than $h$, it indicates successful detection of the watermark flow; otherwise, it indicates unsuccessful detection. We conducted multiple experiments and recorded the watermark detection success rate to reflect the watermark's robustness.

**Figure 7:**
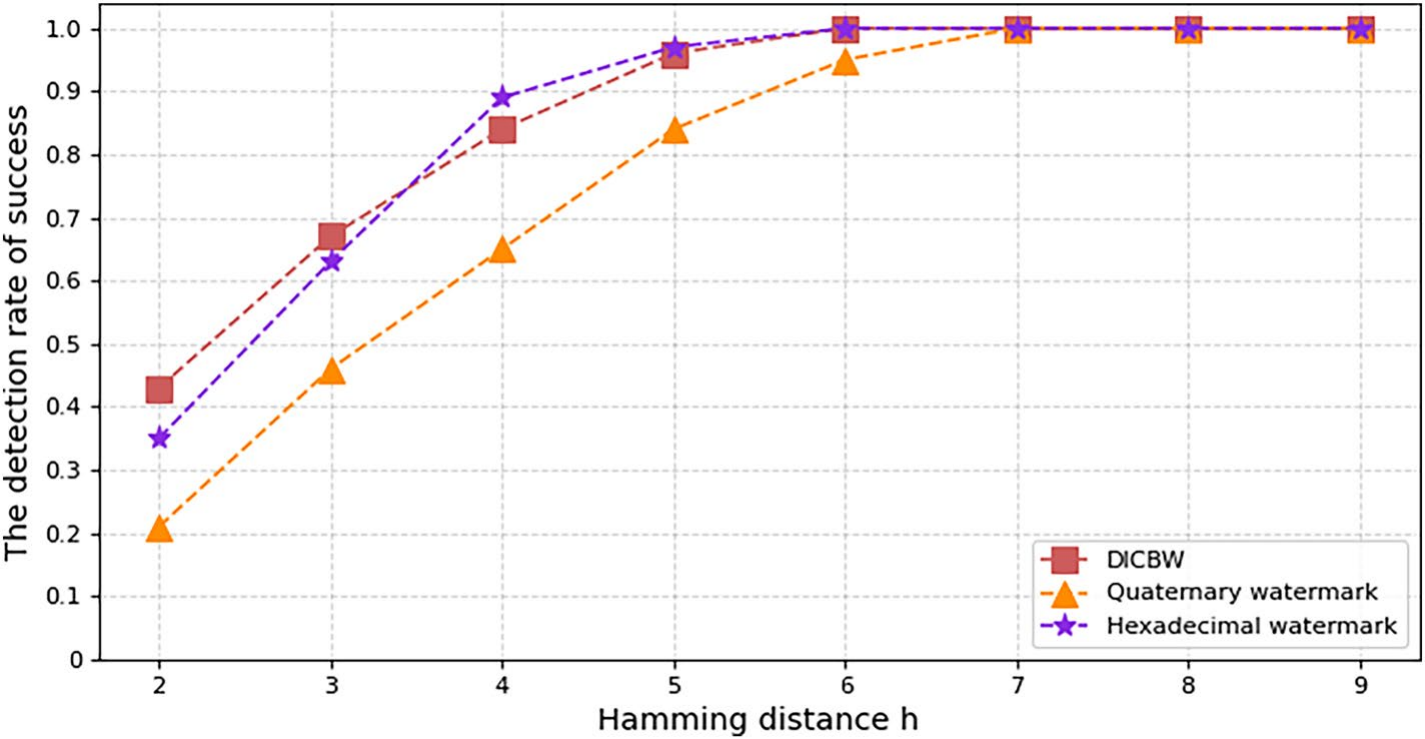
The detection success rate of watermark with different Hamming distances.

Firstly, we considered the impact of the Hamming distance on the watermark detection success rate. The experimental parameters were set as follows: the original watermark length was 60 bits, the network flow speed was $V_{pps} = 10pps$, the interval length of DICBW and efficient quaternary network flow watermark was set to 2 s, and the network jitter was 100 ms. We recorded the watermark detection success rate for Hamming distances $h$ from 2 to 8. As shown in Fig. 7, as the Hamming distance $h$ increases, the watermark detection accuracy also increases. When $h = 6$, the watermark detection success rate of the three NFW schemes is all above 95%, and the detection success rate of DICBW and efficient hexadecimal network flow watermark method is close to 100%.

To test the robustness of the proposed NFW scheme against network disturbances, we conducted the following experiment. The original watermark length was set to 60 bits, and the network flow speed was $V_{pps} = 10pps$. The interval length of the DICBW and efficient quaternary network watermarks was set to 2 s. An interference generator was placed between the watermark embedding and detection devices to produce random delays that mimic real network disturbances. The disturbances were increased from 0 to 250 ms in 25 ms increments. The experiment recorded the changes in the detection success rates of the three network watermarks as the network disturbances increased, with Hamming distances of $h = 3$ and $h = 6$.

**Figure 8:**
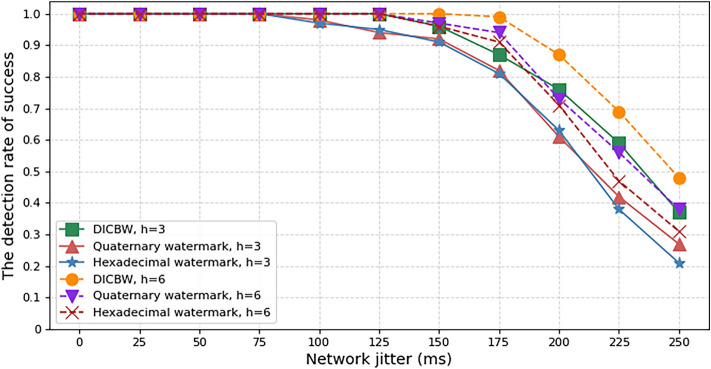
The detection success rate of watermark with different network jitter.

The experimental results are shown in Fig. 8, when the network disturbance was below 100 ms, all three network watermark schemes could ensure a watermark detection success rate of over 95%. When $h = 3$, the detection success rates of the three network watermarks were all above 90% when the network disturbance was 150 ms. When $h = 6$, the detection success rates of the three network watermarks were all above 90% when the network disturbance was 175 ms. The experiment verified that the hexadecimal network watermark proposed in this scheme has slightly lower robustness against network disturbances than the DICBW scheme. However, it has similar resistance to disturbances as the quaternary network watermark and is highly resistant to disturbances, making it suitable for network environments with network disturbances.

In a real network environment, network jitter is not the only type of interference that may occur. Other types of interference, such as packet loss and false packet injection, can also occur due to poor network conditions or malicious attacks. To evaluate the impact of packet loss and false packet injection on network flow watermark, this study conducted experiments with an interferer that simulated a real network communication environment. The experimental parameters included an original watermark length of 60 bits, a network flow rate of $V_{pps} = 5pps$, a network jitter of 100 ms, and interval lengths of 2 s for both DICBW and efficient quaternary network flow watermark. The packet loss rate was $r_{loss}$, and the false packet injection rate was $r_{insertion}$. Two sets of experiments were conducted for $r_{loss} = r_{insertion} = 10\%$ and $r_{loss} = r_{insertion} = 20\%$, with the Hamming distance $h$ increasing from 1 to 9, and the detection success rate of the watermark was recorded.

**Figure 9:**
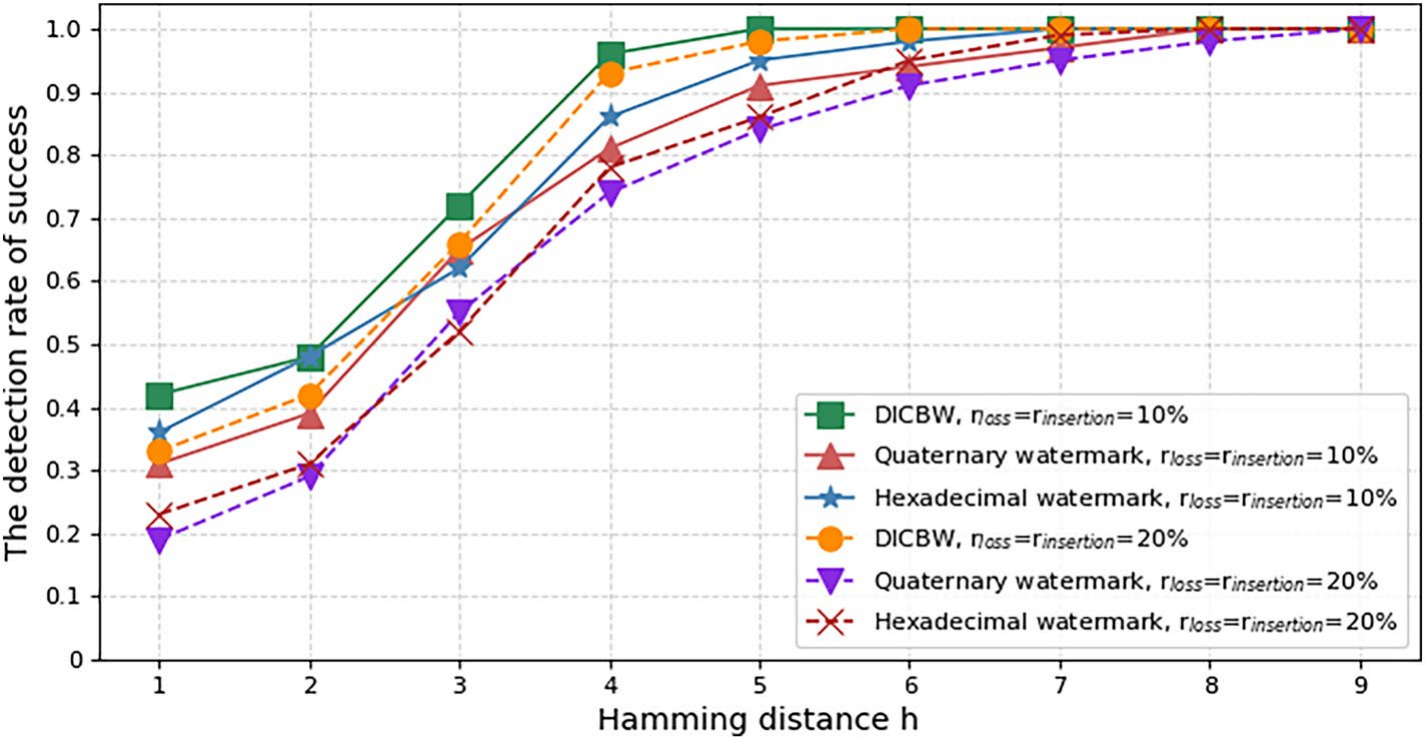
The detection success rate of watermark with different packet loss rate and false packet insertion rate.

The experimental results shown in Fig. 9 indicate that the horizontal axis represents the Hamming distance $h$, and the vertical axis represents the detection success rate. It can be observed that when $r_{loss} = r_{insertion} = 10\%$ and the Hamming distance $h$ is set to 5, the detection success rate of the proposed efficient hexadecimal network flow watermark method is over 95%. Similarly, when $r_{loss} = r_{insertion} = 20\%$ and the Hamming distance $h$ is set to 6, the detection success rate of the efficient hexadecimal network flow watermark method can also reach over 95%. As shown in figure, compared with the DICBW and quaternary network flow watermark schemes, the proposed hexadecimal network flow watermark scheme has slightly lower robustness in the presence of network jitter, packet loss, and false packet insertion, but higher robustness than the efficient quaternary network flow watermark scheme. This scheme has good anti-interference ability and can ensure high robustness even in harsh network environments.

### Man-in-the-middle attack identification

In various network attacks, a man-in-the-middle attack stands out as a commonly employed method. This type of attack occurs when an assailant intervenes in the communication process, misleading both communicating parties into believing they are directly communicating. In reality, all communications are monitored and manipulated by the attacker. Such deceptive actions involve masquerading as a legitimate participant in the communication, allowing the attacker to perform malicious operations. Consequently, man-in-the-middle attacks can be regarded as a specific manifestation of network identity deception.

Based on the severity of the man-in-the-middle attack, it can be classified into two categories: flow monitoring man-in-the-middle attack and flow tampering man-in-the-middle attack. These categories correspond to the attack effects of monitoring and tampering with network traffic, respectively. In a man-in-the-middle attack, data from both communicating parties are intercepted, completing the identity deception. This process alters the timing characteristics of the original network flow, thereby impacting the effectiveness of network flow watermark detection. The efficient hexadecimal network flow watermarking scheme proposed in this paper addresses the issue by determining the presence of a man-in-the-middle attack in the network through the success rate of watermark detection. This approach serves as a defense against network identity spoofing and effectively resolves the problem of man-in-the-middle attacks.

**Figure 10:**
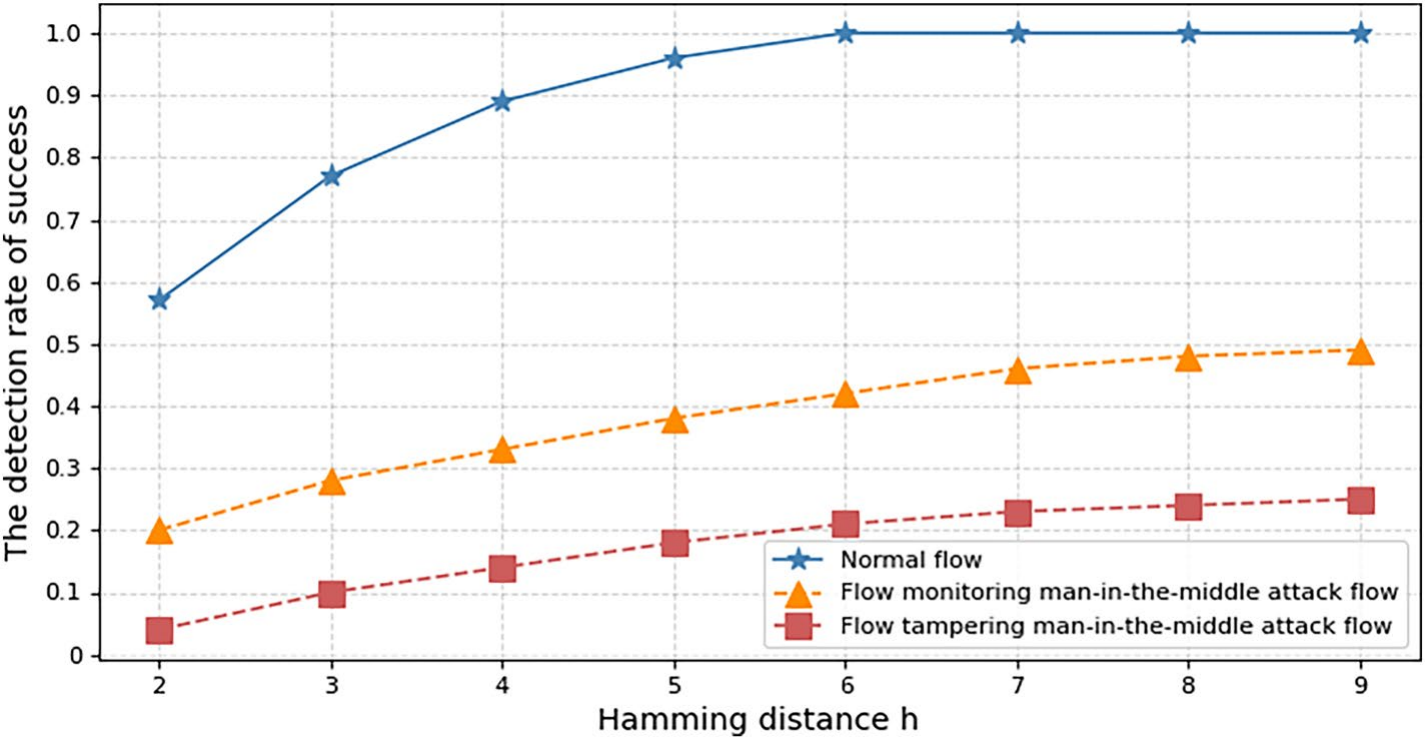
Impact of man-in-the-middle attacks on detection success rate.

To verify the detection of man-in-the-middle attack by efficient hexadecimal network flow watermark scheme, the following experiments are conducted. The jammer is used for packet monitoring, tampering, and resending to simulate the man-in-the-middle attack process. The experimental parameters are set as follows, the length of the original watermark is 60bit, the network flow rate $V_{pps} = 10\,pps$, the network jitter is 100 ms, and the experiments recorded the success rate of watermark detection for normal flow, stream monitoring man-in-the-middle attack flow, and flow tampering man-in-the-middle attack flow as the Hamming distance $h$ varied from 2 to 9, the experimental results are shown in Fig. 10.

From the figure, it can be seen that when the Hamming distance $h$ is greater than 4, the success rate of watermark detection is higher than 90% in a normal network without man-in-the-middle attack, whereas the success rate of watermark detection is not higher than 50% in a network with flow monitoring man-in-the-middle attack, and not higher than 25% in a network with flow tampering man-in-the-middle attack.

The experiment proves that the man-in-the-middle attack has a great influence on the detection success rate of watermark in network flow. By setting appropriate watermark parameters, the efficient hexadecimal network flow watermark scheme proposed in this paper has a good effect on the detection of man-in-the-middle attacks and can effectively solve the network identity deception problem.

## Conclusion and future work

In this paper, we propose an efficient hexadecimal network flow watermark method and introduce the concept of “watermark capacity” to enhance the watermark capacity of network flows through the scheme. Based on the high efficiency of the currently commonly used interval packet counting-based network flow watermark, this scheme divides a single interval into six sub-intervals and embeds watermark information by modulating the packet distribution within each sub-interval. Each sub-interval can modulate 4-bit watermark information, significantly improving the encoding efficiency of the watermark and reducing the time overhead of watermark encoding. We also propose a watermark start mark and watermark end mark, and self-learning watermark interval length by network stream features to further improve the practicality and robustness of the watermark. We conduct experiments on real network flows (including ICMP traffic and SSH traffic) to verify that the efficient hexadecimal network flow watermark method greatly improves the encoding efficiency and watermark capacity of network flows while ensuring anti-interference and robustness. And it can be very effective at detecting man-in-the-middle attack problems and resisting network identity spoofing.

However, there are still some issues with this scheme, mainly in terms of the concealment of network flow watermark. The watermark embedding changes the packet distribution of network flows to some extent, resulting in poor concealment of the watermark, making it easy for watermark attackers to distinguish between watermark flows and normal flows, and therefore discover and destroy watermark information. Therefore, improving the concealment of the watermark in this scheme is urgently needed in future work. Additionally, applying the hexadecimal network flow watermark to high-rate network flows is still a challenging task. These issues will be considered for improvement in the future.

## Data Availability

The experimental packet dataset is available at http://www.caida.org/data/passive/passive_2015_dataset.xml.
